# Dietary Supplementation of Honey Bee Larvae with Arginine and Abscisic Acid Enhances Nitric Oxide and Granulocyte Immune Responses after Trauma

**DOI:** 10.3390/insects8030085

**Published:** 2017-08-15

**Authors:** Pedro Negri, Leonor Ramirez, Silvina Quintana, Nicolás Szawarski, Matías Maggi, Yves Le Conte, Lorenzo Lamattina, Martin Eguaras

**Affiliations:** 1 Centro de Investigación en Abejas Sociales (CIAS), Universidad Nacional de Mar del Plata (UNMdP), Dean Funes 3350, Mar del Plata CP 7600, Argentina; n.szawarski@gmail.com (N.S.); biomaggi@gmail.com (M.M.); mjeguaras@gmail.com (M.E.); 2 Consejo Nacional de Investigaciones Científicas y Técnicas (CONICET), Buenos Aires, Godoy Cruz 2290, Argentina; lramirez@mdp.edu.ar (L.R.); biologiamolecular@farestaie.com.ar (S.Q.); lolama@mdp.edu.ar (L.L.); 3 Instituto de Investigaciones Biológicas (IIB-CONICET), UNMdP, Dean Funes 3350, Mar del Plata CP 7600, Argentina; 4 Laboratorio de Biología Molecular, Farestaie, Mar del Plata CP 7600, Argentina; 5 INRA Centre de Recherche Provence-Alpes-Côted’Azur, Unitè Abeilles et Environnement, UMR PrADE, Domaine Saint Paul, Site Agroparc, Avignon F-84914, France; yves.le-conte@inra.fr

**Keywords:** *Apis mellifera*, abscisic acid, nitric oxide, immune response

## Abstract

Many biotic and abiotic stressors impact bees’ health, acting as immunosupressors and contribute to colony losses. Thus, the importance of studying the immune response of honey bees is central to develop new strategies aiming to enhance bees’ fitness to confront the threats affecting them. If a pathogen breaches the physical and chemical barriers, honey bees can protect themselves from infection with cellular and humoral immune responses which represent a second line of defense. Through a series of correlative studies we have previously reported that abscisic acid (ABA) and nitric oxide (NO) share roles in the same immune defenses of *Apis mellifera* (*A. mellifera*). Here we show results supporting that the supplementation of bee larvae’s diet reared in vitro with l-Arginine (precursor of NO) or ABA enhanced the immune activation of the granulocytes in response to wounding and lipopolysaccharide (LPS) injection.

## 1. Introduction

Honey bees (*Apis mellifera*) contribute with the pollination services which are vital to the maintenance of our ecosystem and stability of many crop yields [1]. Since bee pollinators are important for crop production, high annual losses of managed honey bees achieved great public and scientific concern [2]. Consequently, scientists have dedicated much of their latest work to uncovering the stresses affecting honey bees [2].

Several studies have focused on assessing the relationship between colony health and the effects of multiple biotic (parasites and pathogens) and abiotic (agrochemical exposure and low temperature) stressors [2,3]. The immunosuppression induced by the factors named above has been linked to the bees’ immune deficiency which is assumed to be implicated in colony losses [3]. The stated above highlights the importance of studying the immune response of honey bees. Particularly, understanding the cellular components of the immune system is central to the search of new strategies to enhance bees’ fitness [4].

Honey bees have several lines of innate immune defense against parasites and pathogens [5]. Physical and chemical barriers are a first line of defense that prevent invaders from adhering to or entering the body [6,7,8]. If a parasite or a pathogen breaches those barriers, honey bees can protect themselves from the invasion by activating cellular and humoral immune responses (which represent a second line of defense) [6,7,8].

Nitric oxide (NO) is a highly reactive multifunctional free radical generated during the oxidation of l-Arginine to l-Citrulline by the enzyme NO synthase (NOS) [9]. Numerous reviews have described central roles for NO signalling in host defense mechanisms against infections caused by viruses, bacteria, and protozoan and metazoan parasites [9,10]. In insects, NO is produced as an immune effector molecule in response to microbial infection in several species of lepidopterans, hemipterans, and dipterans, functioning both a signalling molecule and a cytotoxic component [10]. Therefore, NO has been proposed as a key molecule to confront parasites in invertebrates [10]. 

Abscisic acid (ABA) is a phytohormone that regulates fundamental physiological functions in plants [11] and whose presence has been unambiguously demonstrated in nectar and honey [12,13,14] as well as in adult honey bees [12,15,16]. In the last decade, evidence has been accumulated demonstrating that ABA operates as a cytokine in animal cells by stimulating innate immune defences such as cell migration and phagocytosis and by inducing NO and reactive oxygen species (ROS) production [17]. 

Through a series of correlative studies concerning honey bees, we have previously reported that:

(1) NO participates in granulocyte spreading [18].
(2) Wounding induces NO production in granulocytes and augmented the total number of granulocytes in larvae hemolymph [19].
(3) ABA supplementation in the syrup of honey bee colonies enhanced granulocyte spreading [15].
(4) ABA supplementation enhanced the number of granulocytes (which are the NO-producing hemocyte type described in *A. mellifera*) in larvae hemolymph after wound challenge [15].
(5) ABA supplementation enhanced wound healing in larvae, reversing the anticoagulant effects of *Varroa destructor* (*V. destructor*) parasitism [15].
(6) ABA supplementation enhanced phenoloxidase activity in adult bees [15].
(7) ABA supplementation enhanced colony survival through winter in field approaches [15] and recently we have reported that ABA enhances cold tolerance in honeybee larvae [16].

As described above, we have previously reported that NO and ABA play roles in the activation of the cellular immune defense of *A. mellifera*. The series of correlative work cited above allowed us to consider cellular spreading and production of NO as indicators of immune activation of *A. mellifera* granulocytes. In this work, we aimed to study if the cellular immune could be enhanced through dietary supplementation with l-Arginine (precursor of NO) or ABA in in vitro-reared honey bee larvae.

## 2. Materials and Methods 

### 2.1. In Vitro Rearing of Bees 

One day old *Apis mellifera* (*A. mellifera ligustica–A. mellifera mellifera*) larvae were transferred from brood comb to a queen rearing cup and the cups were placed into 48-well culture plates. The larval rearing plates were placed into desiccators maintained at a relative humidity of 96% (K_2_SO_4_-saturated) and 34 °C into an incubator. 

The larvae were fed with a diet containing 50% of royal jelly, obtained from a commercial supplier, and 50% of sugar solution composed by yeast extract, D-glucose (Sigma-Aldrich, St. Louis, MO, USA), and D-fructose (Fluka, St. Gallen, Switzerland). Daily diet volumes provided to the larvae and variation of the composition of the diet was done according to Aupinel et al. [20] (see also Crailsheim et al. [21]). All larvae received a total of 160 mL diet during the six-day rearing period.

In each independent experiment, a group of larvae were fed with the standard diet (control), another group of larvae were fed with the standard diet supplemented with l-Arginine (l-Arg, 5 mM), and another group of larvae were fed with the standard diet supplemented with ABA (ABA, 50 µM).

### 2.2. Lipopolysaccharide Challenge of in Vitro-Reared A. mellifera Larvae

In each independent experiment, the larvae from each diet group (control, l-Arg, and ABA) were immunologically challenged and evaluated as follows when they reached the first day of the fifth instar (L5).

Three larvae from each diet group (control, l-Arg, and ABA) were selected and injected with one µL of sterile culture medium (Excell-405) alone (control of injection).

Another three larvae were selected from each diet group and injected with 1 µL of sterile culture medium (Excell-405) containing lipopolysaccharide (LPS 1 mg/mL). Lipopolysacharide is a classic inducer of NO production involved in innate immune responses in vertebrates, invertebrates, and even in plants [22,23,24].

Three larvae from each diet treatment (control diet, l-Arg-supplemented, and ABA-supplemented) were selected as control individuals (naïve) without being injected.

The injections were performed using a Hamilton syringe (10 µL) with a fine needle (34 gauge tip style four, Hamilton). 

Twenty-four hours after the microinjections had been performed, all the injected larvae (control injected and LPS) were evaluated according to their appearance and only the larvae showing a healthy phenotype and with healed wounds were selected for further analyses. The larvae that were not selected for the experiments were discarded. Finally, two larvae were selected per group in each independent experiment gaining a total of four larvae evaluated for each of the nine experimental groups.

Following the selection of the larvae, the hemolymph from the selected larvae was extracted and the immune activation (cellular spreading) and the production of nitric oxide (NO) were analyzed in each granulocyte observed within each hemocyte culture.

The reason for having selected only two larvae from each diet group reduced the number of cell cultures to be observed under the microscope and this enabled an accurate evaluation of the cells. The granulocytes of each replicate were photographed and analyzed for cellular spreading and NO production (as described below). The data obtained from the four larvae of each of the nine experimental groups from the two independent experiments were pooled.

A total of 64 granulocytes evaluated from larvae fed with the control diet but that had not been subjected to any trauma (not-injected larvae); 93 granulocytes evaluated from the larvae fed with the l-Arginine-containing diet but that had not been injected; 62 granulocytes evaluated from larvae fed with the ABA-supplemented diet but that had not been challenged with any trauma (not-injected larvae); 71 granulocytes evaluated from blood extracted from larvae fed with the control diet that had been subjected to the injection trauma alone (injected without LPS); 76 granulocytes evaluated from the larvae fed with the l-Arginine-containing diet that had been injected with the media alone (injected without LPS); 64 granulocytes evaluated from larvae fed with the ABA-supplemented diet that had been challenged with the injection of media without LPS; 64 granulocytes evaluated from larva fed with the control diet and injected with LPS; 53 granulocytes evaluated from larvae fed with the l-Arginine-supplemented diet and that were also injected with LPS; 53 granulocytes evaluated from larvae fed with the ABA-containing diet and injected with LPS.

### 2.3. Hemolymph Collection

Hemolymph collection was performed as previously described by Negri et al. [8,15,18,19]. Briefly, insect blood was collected from fifth instar larvae by puncturing the soft cuticle with a sterile 30 G needle. Before puncturing, the cuticle was surface sterilized with 70% ethanol. The hemolymph was collected with a micropipette and transferred to a microcentrifuge tube containing Excell-405 insect cell culture media (Sigma). Hemolymph solution was homogenized by soft pipetting into the sampling tube and then transferred to sterile 96-well glass bottom plates (NUNC, 96-well optical bottom plates, sterile) where all the hemocyte behavior parameters (NO production, spreading, and types of adherent hemocytes) were analyzed. Equal volumes of hemolymph were extracted from the control and the injected larvae (15 μL). 

### 2.4. Nitric Oxide Detection and Quantification

The endogenous synthesis of NO in living hemocytes was evidenced as in Negri et al. [18,19] by means of the fluorescent probe DAF-FM DA (Molecular probes, Invitrogen, Waltham, MA, USA) at a final concentration of 10 μM in the culture media. This dye emits green fluorescence within cells after reacting with NO. To measure the NO production in the hemocytes, pictures were taken and the green fluorescence emitted by the cells was quantified using ImageJ (National Institute of Health, Bethesda, MD, USA, public domain software). The observations were made at room temperature. This has been proven to be a reliable process which can obtain quality measurements for NO production within *A. mellifera* hemocytes cultured in vitro [18,19].

### 2.5. Hemocyte Spreading Evaluation

Cells that attached and spread upon stimulation with the glass surface were considered immune-related hemocytes [8,18]. Hemocyte count using a hemocytometer was avoided to reduce the manipulation of the hemocytes to be analyzed in the assays. Cellular spreading of each granulocyte was measured using ImageJ [8,15,18,19].

### 2.6. Microscopy

After being transferred to the 96-well plates, the mixtures containing *A. mellifera* hemolymph were observed using inverted microscopy. Hemocytes were examined by means of differential interference contrast (DIC) or epifluorescence (excitation at 480 nm–emission at 520 nm) microscopy. The microscope used was a Nikon Eclipse *Ti* inverted microscope using a 60× objective.

### 2.7. Statistical Analysis 

Statistical analyses were conducted with SigmaPlot v. 11.0 software (Systat Software Inc., San Jose, CA, USA). Data from cellular area quantification and NO production (green fluorescence levels) were analyzed using the Kruskal–Wallis method (*p* < 0.001) followed by the method for multiple comparisons of Dunn (*p* < 0.05) due the assumptions of normality and/or homogeneity of variances were violated. 

## 3. Results

The effects of l-Arginine and ABA dietary supplementation over granulocyte spreading and NO production were evaluated in response to LPS-injected in in vitro-reared *A. mellifera* larvae.

**Figure 1:**
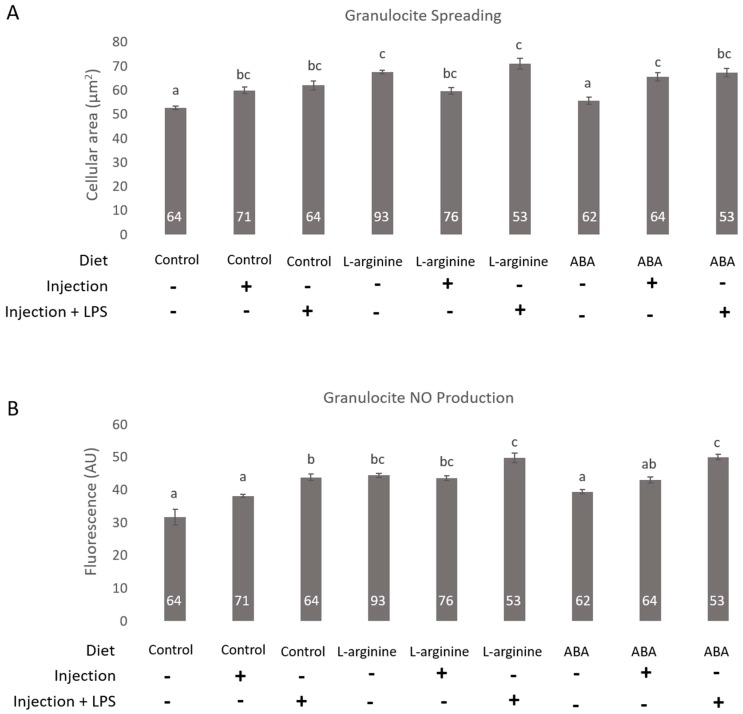
Effects of dietary l-Arginine and abscisic acid (ABA) over granulocyte spreading and NO production in response to lipopolysaccharide (LPS) in in vitro reared *Apis mellifera* larvae. Control: Larvae fed with the standard diet for in vitro rearing; l-Arginine: Larvae fed with the standard diet supplemented with 5 mM l-Arginine; ABA: Larvae fed with the standard diet supplemented with 50 µM ABA. LPS: lipopolysaccharide (1 mg/mL). (**A**) Cellular spreading was measured as cellular area (mean ± SE) using ImageJ software from pictures obtained with a photographic camera coupled to the microscope; (**B**) NO production was measured using ImageJ from the green fluorescence emitted by the photographed hemocytes and expressed in arbitrary units (AU, mean ± SE). A Kruskal–Wallis analysis was applied (*p* < 0.001) followed by the post hoc method for multiple comparisons of Dunn (*p* < 0.05). Different letters means significant differences between treatments (*p* < 0.05). The number of granulocytes evaluated are detailed in white numbers on the bases of the black bars.

As Figure 1A shows the injection alone and the injection + LPS induced the spreading of the granulocytes of the control larvae (Figure 1A). The production of NO in the granulocytes was significantly enhanced also by LPS treatment in the control group (Figure 1B). 

l-Arginine supplementation enhanced both granulocyte spreading and NO production when larvae had not been subjected to any challenge compared to the control group (Figure 1A,B). In response to injection, the l-Arginine-supplemented larvae showed significantly different levels of NO production compared to the control group (Figure 1A). The granulocytes from the l-Arginine-supplemented larvae showed enhanced cellular spreading and NO production in response to LPS challenge compared to the control group (Figure 1A,B).

The granulocytes from ABA-fed larvae showed stronger spreading in response to LPS treatment compared to the control group (Figure 1A). The production of NO was enhanced in response to injection alone and and LPS challenge in granulocytes from the ABA supplemented larvae compared to the control group (Figure 1B). 

## 4. Discussion

In this work, an in vitro rearing system was used to provide with new evidence supporting the idea that dietary l-Arginine could work systemically as the source for NO production in honey bees. At the same time, new data reinforcing the systemic effect of ABA over the cellular immune response in *A. mellifera* is shown.

It is important to highlight that the extraction of hemolymph is an immune challenge per se. In combination with the challenge of the hemocytes with foreign non-self surfaces like glass, this had been the immune stimulation used in previous reports in *A. mellifera* [8,18]. Here, the aim of the experimental design was to induce the immune stimulation within the living larvae and then evaluate the efffect of the immune challenge in vitro. 

Here, we considered the cellular spreading and the production of NO as indicators of immune activation of *A. mellifera* granulocytes. We focussed on evaluating those responses in granulocytes backed-up by the series of correlative work reported previously [8,18,19] supporting that these are the cells that first react in response to immune challenge. Future studies should include the evaluation of other hemocyte types as well. 

Through the evaluation of the cellular response to injection and LPS observed in the control group, it could be speculated that the injection per se induced the immune activation of the granulocytes. This could be related with the wound generated by the needle as it had previously been reported in Negri et al. [15,19].

As it has been previously described [8], the challenge with LPS induced the spreading response in all the treatment groups. Here the results indicated that LPS also induced NO production in granulocytes of *A. mellifera* as it has been reported in other biologycal models [22,23,24]. Thus, it could be assumed that the cellular immune response was activated by wounding and bacteria-related challenge and that NO plays a role in that process in *A. mellifera* larvae. These results are in concordance with previous reports by Negri et al. [8,15,18,19]. 

The aminoacid l-Arginine is the substrate used by the enzyme nitric oxide synthase to produce NO. Here, the granulocytes extracted from the larvae that had been fed with a diet supplemented with l-Arginine showed an augmented production of NO production. At the same time, the results showed here support that both granulocyte spreading and NO production responses were enhanced by dietary l-Arginine in *A. mellifera* larvae. These results coincide with previous reports describing that NO participates in granulocyte activation and wound response in Drosophila melanogaster [25,26] and *A. mellifera* [18,19]. 

In the present work, the response to LPS was enhanced by dietary ABA in honey bee larvae. At the same time, while the control diet group did not show an enhanced NO production in response to injection alone, the granulocytes from the ABA-supplemented larvae evidenced a significantly augmented synthesis of NO as it was also observed in l-Arginine-fed larvae. 

The results described above are in concordance with previous reports indicating that ABA treatment enhanced cellular responses in *A. mellifera* larvae in response to wounding [19]. At the same time, the results showed here support the idea that ABA could enhance the NO-mediated inmune response to LPS of honey bees as it has been demonstrated in other animal models [17,27]. However, more experiments should be performed to get an accurate description of the mechanisms behind these responses. 

All in all, the results discussed above represent new evidence supporting the roles of ABA and NO in *A. mellifera* immune defenses. However, more experiments should be performed to reinforce the results obtained here.

The search for relevant immunomodulatory tools to enhance the immune strength of honey bees represents a promising way to improve their resilience to environmental stress [28]. In this sense, ABA and NO should be taken into consideration as key molecules to improve individual immune defenses in honey bees. Abscisic acid and l-Arginine could be promising nature-derived dietary supplements to enhance the fitness of honey bee colonies and could be easily coupled in the beekeepers’ practices.
